# Additional Reports on the Effects of a Peculiar Regimen in Cases of Cancer, Scrofula, Consumption, Asthma, and Other Chronic Diseases

**Published:** 1816-11

**Authors:** 


### PART II.

### COMPREHENSIVE ANALYTICAL REVIEW OF MEDICAL LITERATURE.

"Tros, tyriusve, nobis nullo discrimine agetur."

*Additional Reports on the Effects of a peculiar* Regimen in Cases of Cancer, Scrofula, Consumption, Asthma, and other Chronic Diseases.* By WILLIAM LAMBE, M. D. 1 vol. 8vo. pp. 526. London, 1815.

THAT death is the common lot of all mankind, cannot be denied; but that its *premature* accession, by the long black catalogue of diseases, is owing to the tenderness, the luxury, and corruptions introduced by the vices and false refinements of civil society, has been the opinion of many able philosophers, both amongst the ancients and the moderns.

"That delicacy which is injured by every breath of air, and that rottenness of constitution, which is the effect of indolence, intemperance, and debauchery, were never intended by the Author of Nature; and it is impossible that they should not lay the foundation of numberless sufferings, and terminate in premature and miserable deaths."

When we consider the beautiful symmetry of the human frame, the admirable construction and adaptation of its different parts, and the various functions by which it is preserved, it certainly appears strange, that this masterpiece of the creation should be so liable to derangement and disease! More than half the human race perish in infancy; and of the remainder, a large portion are the victims of pain and suffering. Even among the apparently strong and healthy, if we examine narrowly into their habits and feelings, we shall hardly find an individual who will not acknowledge some defect, some secret uneasiness; something that diminishes his present comfort, and which excites apprehension for the future!

* Price on Reversionary Payments.

"The moral traits are as much distorted as the physical. The affections, which should link man to man, and make each human being regard his fellow creature as his brother, are choked and almost extinguished. Envy, hatred, jealousy, and all the malignant passions, predominate in the human bosom. The infliction of pain upon sensitive beings, instead of exciting compassion, is, with the multitude, a source of pastime and merriment. To such a degree are the strongest instincts of our nature perverted, that the first principle of self preservation is finally destroyed; the hand is raised against the existence of its possessor; or the parental arm against the life of the offspring." p. 44.

But we must confine ourselves to physical evils. If we survey the surface of the globe, we shall perceive tribes of diseases, which are dreadfully fatal, domiciliated, as it were, in certain situations. We have only to instance Batavia; the West Indies; Walcheren; the Pontine Marshes, &c. &c. when a thousand other places will occur to the mind. Now it is quite evident, that the endemic diseases peculiar to these soils, and which have swept away, and are still sweeping, large portions of the human race to an untimely grave, are not occasioned by any inherent debility or defect in the human constitution, but by the operation of extraneous and foreign causes. The same may be said of the various contagious diseases, as plague, typhus, variola, measles, chincough, scarlatina, syphilis, &c. These are, surely, factitious, and the product of society; not an essential condition annexed to the existence of man. Scurvy is another instance of artificial disease. The goitre or bronchocele is clearly a local affection, almost peculiar to the vallies of mountainous countries, where the heat is concentrated, the air stagnant, and the water impregnated. To the same causes may be ascribed *cretinism,* that most deplorable of human infirmities.

"As, then, a large portion of the diseases, which cut men off in civil society, are proved to be artificial; and as there is reason to suspect the same thing of others, in which so direct a proof cannot be obtained; there can be nothing extravagant or absurd in the supposition, that they are all artificial, and to be traced to some morbific causes, either of circumstances or manners. And should this hypothesis, after due investigation, prove correct, there can be nothing absurd, extravagant, or enthusiastic, in the hope, that, finally, successful methods may be discovered either of treating them when formed, or, at least, of preventing their formation." p. 61.

From Dr. Lambe's 4th Chapter, we shall extract some interesting particulars relative to the comparative mortality of the human race, as influenced by locality, and other accidents of life, foreign to the body itself.

The proportion between the whole numbers living at any age and upwards, and the whole number of the community, is a fixed proportion. And from this, the havoc made in human life, by collecting multitudes of men together in great cities, is fully demonstrated. In London, half the number born die under three years. In Vienna and Stockholm, under two. In great towns about $\frac{1}{20}$th of the whole population die annually. In moderate towns, about $\frac{1}{25}$th. In the country, from $\frac{1}{35}$th to $\frac{1}{60}$th. In London, the probability at birth is twenty years. At Holy Cross, near Salop, thirty-three years. At Ackworth in Yorkshire, half the inhabitants born, live to the age of 46. In the town of Manchester, the annual mortality is 1 in 28. In the country contiguous, 1 in 56! At Holy Cross, *half* the inhabitants die above 80. At Northampton, 1 in 22 reaches this age. At Norwich, 1 in 27. In London, 1 in 40. Of those *born* in London, not 1 in 60 attains an octogenarian age! *Price on Reversionary Payments.*—From these and other documents, Mr. Malthus, as well as Dr. Price, was led to the following conclusion ;

"Diseases," says he, "have been generally considered as the inevitable inflictions of Providence ; but perhaps the greater part of them may more justly be considered as indications that we have offended against some of the laws of Nature."

Dr. Lambe is of opinion, that the four principal circumstances which contribute (independently of the contagions) to the generation of constitutional diseases, are, impure air ; impure water ; improper aliment ; fermented liquors. On air he has nothing to say. On water he has nothing to add to what he has already advanced in his former work.

"Some observations on the utility of vegetable regimen ; the mischiefs of the regimen in common use ; and a few remarks on fermented liquors, is all that he proposes to add to the introductory part of his present undertaking." p. 88.

It is very generally believed, that *habit* will reconcile us to any thing, and counteract the baleful influence even of poisonous substances. But this is a mistake, for sooner or later we feel the effects of aberrations from the dictates of Nature. Dr. L. considers *animal food* as one of those "habitual irritations" which, at least, predispose to various diseases. We shall endeavour to condense and convey our author's meaning in our own language.

The Negroes of the West Indies are exempt from the yellow fever; they live almost exclusively on *vegetables.* The same class in North America were found susceptible of the causes of this fever; they live much on *animal* food.* Among the White's, the French, who use less meat than the English, in Tropical Climates, are less affected by the endemic fevers. At Constantinople, the Armenians, (an herbivorous race) are far less disposed to the plague than other people, according to the testimony of Timoni. When typhus attacks the poor and the rich, the latter have a worse chance of recovery, if treated in a similar manner. The vegetable eaters of the Eastern world recover sooner from severe wounds than Europeans. For the truth of this, we can vouch from personal observations. On this principle may partly be explained the superior salubrity of the *country,* where the same classes of people eat less animal food than in large towns. Multitudes of rich valetudinarians have been restored to health by adversity. Dr. Berwick states, that his brother, who was confined in the Tower several years on bread and water, laboured under *phthisis* before his captivity, but came out sleek, plump, and gay. Ramazzini relates the case of a man who had been a victim to gout, but was completely cured by nineteen years' imprisonment on bread and water. *Opera, p.* 478.—The noble Francis Pechi, ætat. 50, and a martyr to gout, was secretly thrown into a prison, and detained there twenty years on low diet. The French, who took the citadel where he was confined, released him, when, to the astonishment of the inhabitants of Vercilli, the venerable *septuagenary* walked forth through the streets without a stick, and free from stiffness in any of his joints. The miserable victims of elephantiasis were often, in times of old, deserted by their dearest friends, and banished to the wilderness, there to perish in solitude. Aretæus has recorded, that some of these sufferers were restored by the efforts of Nature alone; consequently, where they lived entirely on herbs. Since fishing has declined among the inhabitants of the Ferro Isles, elephantiasis has ceased. Galen ascribes the prevalence of elephantiasis among the poor of Alexandria to the habitual use of salted meats. The well known history of Wood, the miller, of Billericay, who from gluttony and intemperance fell into obesity and disease, but who

* Here we cannot entirely coincide in our author's inference. We consider acclimation as the cause of this difference of susceptibility in the Negroes of the two places.—Edit.

lost all his complaints by vegetable regimen, is in favour of Dr. Lambe's principle. Ulloa testifies, that the inhabitants of South America are remarkable for longevity—and vegetable food. Humboldt confirms this.

"It is the mountainous and barren districts, where frugality and simplicity of manners are the necessary habits of the bulk of the community, that have ever been the favourite abodes of health and longevity."

Examples of longevity are chiefly to be found among the peasants, philosophers, hermits, and anchorites, who have practised abstemiousness from necessity or principle. The members of those monastic orders who abstained from animal food, enjoyed in consequence a longer mean term of existence. The author of "*Apologie du Jeune*," extracted from Baillot the length of the lives of one hundred and fifty-two monks or of bishops, using the same austere life, taking them promiscuously as they presented themselves in all times and climates. They averaged seventy-six years three months. He took one hundred and fifty-two academicians in the same manner, but they only averaged sixty-nine years two months, giving full seven years advantage to the anchorites.

Dr. Lambe has somewhat unfortunately instanced the Bramins as affording examples of longevity. But we know from actual observation, that the range of human existence is more circumscribed in Hindostan than in England; and that instances of longevity are rare indeed in the Eastern world. In support of this, we shall quote the authority of Captain Williamson, a most intelligent officer, who resided more than twenty years in various parts of the East.

"Longevity," says he, "certainly is not characteristic of India. Whether this is owing to excessive heat, or the indolence of the upper, and drudgery of the lower classes, it may be difficult to decide; but certain it is, that we rarely see an instance of any one arriving at 60 years of age." *Oriental Field Sports, vol. i, page* 236.

Nor do we attribute this to abstinence from animal food:—No, we are convinced that the range of existence would be still less if the inhabitants changed their vegetable banquets for European dishes. It is the climate that is the chief cause of the phænomenon.

Dr. Lambe is not so enthusiastic or sanguine as to expect the cure of constitutional diseases from a vegetable regimen, but merely that life will be prolonged, the disorder rendered milder, and the feelings more comfortable. We were somewhat surprised at the conclusion of this chapter to find Dr. Lambe depreciating the utility of low regimen in acute diseases.

"Without at all disputing the propriety of this strictness, I must doubt greatly its efficacy; for having seen severe attacks of inflammatory disease where a regimen of this kind had been followed for months and even for years; I cannot but ask what effect can it be supposed to have on the issue of such disease, when resorted to only on the spur of the occasion; and continued for a few days, or, it may be for a few weeks? I question not then, that their issue depends infinitely more upon the antecedent habits, than by any effect of regimen during their invasion." p. 118.

This to us appears strange doctrine, from a disciple of Pythagoras too; but before offering a comment we shall quote one short passage from Dr. Lambe himself.

"Animal food commonly gives a more succulent habit, *a greater fulness*, and at the same time, a higher colour to the face. What can this indicate but an excitation of all the smaller vessels of the face? This excitation cannot be supposed to be confined to the surface of the cheeks; but must extend to all the contiguous parts: *To the internal as well as the external.*" p. 122-3.

Now we would ask, if the total abstraction of the cause of this excitation *during* acute inflammation, be not a very likely mean of arresting the progress of that morbid process? And if it be answered in the affirmative, we maintain that British practitioners, who enforce a more rigid antiphlogistic regimen in acute diseases than Foreigners, are decidedly right in so doing.

### Chap. 6. *On Vegetable and Animal Food, continued.*

Dr. Lambe thinks, that the pallid countenances of children who live on vegetable food, are apt to create unnecessary alarm, and that the high colour of beef-eaters is by no means so indicative of health, as is vulgarly imagined. He represents the use of animal food as giving an unnatural excitation to the brain, the consequence of which is, that all the stages of life are hurried on with an insalutary rapidity. Puberty and the passions are too early developed. In the male they acquire an almost maniacal impetuosity; females, who ought to be nurses, become pregnant, even with the child at the breast;—finally, premature old age usnrps the period of middle life!

As a general rule, it may be asserted, that the florid are less healthy than those who have little colour. An increase of colour has ever been considered as a sign of impending illness. How many with the glow of health on their cheeks are inwardly tabid. How many on the verge of the grave, about to be cut off by an acute illness?

That animal food disposes to corpulency is evident.—

"You may see," says Dr. Arbuthnot, "an army of forty thousand foot soldiers without a fat man; and I dare affirm that by plenty and rest, twenty of the forty shall grow fat."

Corpulency is of itself a species of disease, and a still surer harbinger of other diseases. The corpulent are sleepy, lethargic, short-breathed, and short-lived! It is an error, however, to suppose, that paleness or leanness is a necessary consequence of a vegetable regimen. Many thrive better on vegetable than on animal food. Those people who are perpetually prating about giving *nourishing* diet to the enfeebled and debilitated, would do well to reflect on the maxim of Hippocrates.

"In bodies that are not pure, the more you nourish them, the more you injure them."

Dr. Lambe thinks, that the ancient physicians and philosophers, though less conversant with drugs than the moderns, had a more correct knowledge of the influence of food upon the health, the morals, and the intellect. Socrates, Plato, Zeno, Epicurus, and other masters of ancient wisdom, adhered to the Pythagoræan diet; enjoyed health, and attained old age.

Immodicis brevis est ætas et rara senectus.

The Cynic Diogenes being asked, why the athletæ were the most stupid of men? replied, "because they are wholly formed of the flesh of swine and oxen." — *Diog. Laert. in vita Diogen.*

Speaking of the sensibility of the nervous system, Dr. Lambe adduces the comparative sprightliness of the French and English as resulting from the opposite modes of diet used by the two nations; but we are of opinion, that many other moral and physical causes combine to produce the national distinction alluded to. Our author is, however, of opinion, that animal food is unfavourable to the intellectual powers; since, after a full meal of meat, the mental operations are slow and clouded, while, with the vegetable feeder, it is morning all day long. In answer to Mr. Lawrence's question, in the Article *Man*, Rees's Cyclopœdia:

"Whether it is possible that the species of food which has formed a Fox and a Pitt, can be unfavourable to the production of talent?"

Dr. L. aptly replies—

"Why did not the writer who has used this argument, carry it to its full extent; and prove that a plentiful use of the *bottle* does not injure the intellect? for it is well known, that one of these illustrious men indulged very freely in his daily potations, and that the other was not remarkable for temperance."

Our author shrewdly suspects, that no stronger proofs of the baleful effects of luxury upon the human character can be given than this fact, that the families of the whole body of the British nobility could produce but *one Fox and one Pitt,* to head the conflicting parties of our Senate; and that these illustrious Senators have not left a single inheritor of their talents among the same body!

> "Fat paunches have lean pates, and dainty bits
> Make rich the ribs; but banker out the wits."—*Shakspeare.*

Of all species of animal food, as a principle article of sustenance, Dr. L. thinks FISH is most unfavourable to longevity. In support of this, he quotes "Cheyne on Health," p. 41; and "Van Troil's Letters on Iceland," shewing the diseases, particularly elephantiasis, prevalent among the fish eaters of that dreary climate. The testimony of Mr. Hooker is to the same effect.

"The milk of herbivorous animals is probably that which approaches most nearly in salubrity to pure vegetable matter." 167.

But our author considers the use of milk as a branch of the carnivorous system; and consequently, as unnatural and insalubrious.

So far we were disposed to coincide, in a *considerable degree,* with Dr. Lambe; but we began to fear that a favourite system had carried him beyond the bounds of sober induction, when we perused the following passage:

"If these observations are correct, the fermenting of bread, and the *cookery of vegetables,* are practices adopted by mankind from the same motives: they accommodate the matters to which they are applied, to the fastidious delicacy of our digesting organs; which is effected, however, at some expence of their strengthening and nutritive powers." 187.

In the 7th chapter, Dr. Lambe is evidently hard pushed to maintain his favourite hypothesis, that animal food is unfriendly, and vegetable conducive to longevity. The Laplanders are known to be, from necessity, almost carnivorous. They are asserted to be both healthy and long-lived.

"Tu ducis, (says Linnæus) innocentissimoss tuos annos ultra centenarium numerum cum facili senectute et summa sanitate. Te latent myriades morborum nobis Europœis communes." Flora Lapponica, p. 165.

To invalidate this strong document, Dr. Lambe distrusts the accounts which the Laplanders give of their own age, as they have no "*authentic registers*;" and as Acerbi has depicted them to be "feeble, aukward, and helpless beings." As they use much milk too, their diet cannot be considered as entirely animal.

Next to Lapland, the vegetable supply of Iceland is the most scanty; flesh, fish, and milk being the principal fare. Dr. Holland, who accompanied Sir George M'Kenzie, states, that "a comparison of facts would probably prove that the longevity of the Icelanders rather exceeds than falls short of the average obtained from the continental nations of Europe." Dr. Lambe explains away this assertion by numerical documents, furnished afterwards by the same traveller, but not quite satisfactorily to us.

Dr. Lambe next takes a rapid view of different tribes who live in a particular manner, endeavouring to draw from them arguments in favour of the vegetable regimen. But we fear that he attributes too much to the operation of food, and too little to the influence of other physical and moral causes, in modifying different classes and tribes of the human race. We shall adduce one example:—Dr. Lambe brings forward the inhabitants of Marquesas, and the other Society and Friendly Islands in general, as exhibiting great beauty, and a bodily organization of the highest degree of perfection. They live chiefly upon vegetables. But the natives of *Mallicollo*, offering an aukward exception, inasmuch as they are represented by Cook and Forster *with faces like apes*, Dr. L. remarks,—

"They practice agriculture, but they *probably* depend much upon their bow and arrows for subsistence, since every man had one, and they were very unwilling to part with them." 209.

This is a weak argument, and savours a little of partiality in favour of an hypothesis.

Before concluding the subject, we shall give a short sketch of our author's ideas respecting the progress of society.

In primæval times, the earth, while left to its natural fertility, and covered with immense forests, afforded sustenance and shelter for every species of animals. Like them, MAN would feed upon the substances to which his instinct would direct him, and which his physical powers would enable him to collect. Devoid of clothing, we must suppose him placed between the tropics, where the air is genial and the earth fertile. The same trees that fed him with their fruits would shade him with their branches. As man must have existed some time before the invention of any art, he could not then have lived by prey, since all the animals excel him in swiftness. There is nothing which indicates a natural and radical hostility between man and animals. The latter betray no fear of the former, when not disturbed or hunted; no feeble indication that Nature intended them to live in peace and harmony with each other. Neither could instinct ever prompt man to devour the dead carcases of animals. The sight of them would rather excite horror, compassion, or aversion.

Living wholly on uncooked vegetables, man would never experience thirst. All the liquids which he would use would be furnished by the juices of fruits and esculent plants. It is highly probable, that, under these circumstances, man would arrive at a high degree of physical perfection: he would have a robust frame, great vigour, great activity, and uninterrupted health. Dr. Lambe, however, does not think that this state is comparable to the benefits of civilization.

This state of Nature could not be of long duration. Man is gregarious; he has the will and power of communicating his ideas by the inflections of his voice. Arts would be invented, and ingenuity would come in aid of physical force. The pride of reason and the wantonness of power would extend his dominion, engender artificial wants, and make him the enemy and the tyrant of his more feeble and less crafty companions.

The savage, the pastoral, and the agricultural states, comprehend the principal forms of society. The energies of the savage are absorbed in the search of food. War is necessary for his precarious subsistence; his mind is congenial with his situation. The hostile and furious passions have uncontrolled possession of his soul. He delights in the infliction of wounds and death—a stranger to remorse, pity, or sympathy.—Even love, in his obdurate bosom, is a transient spark! This is a state in which *instinct* is the most completely annihilated, and reason is the most feeble. The condition of the savage, in fact, so far from being natural, is that which recedes farthest from the state of Nature.

By fencing in land, and preserving a portion of the natural herbage, man was enabled to domesticate some tribes of animals, over whom he assumed despotic power. He availed himself of their labour, and applied their milk and flesh to his own sustenance. He became a shepherd, but still civilization was imperfect. The hordes of barbarians who have desolated kingdoms and subverted empires were pastoral tribes.

The cultivation of the earth, and the application of its various productions to human sustenance, seem to be the limit of improvement in the arts essential to the support of life. By these, myriads of human beings are called into life; who would otherwise have slumbered in non-existence. By these, domestic tranquillity is preserved, and the causes of foreign hostility or rancour are removed.

"It seems no visionary or romantic speculation to conjecture, that if all mankind confined themselves for their support to the productions supplied by the culture of the earth, war, with its attendant misery, might cease to be one of the scourges of the human race." 239.

We much fear that the human *passions* must be subdued, before this *millennium* can be hoped for on earth! Can it be expected, that a system of diet calculated to bring the structure and functions of the human body to the greatest possible degree of perfection, would, while it insured health and vigour, restrain those passions which foster hostility and rancour, not only among individuals, but among nations? It must be from a moral rather than a physical regimen, that the blessings of peace and good will on earth can be procured and maintained.

### Chap. 8. *On the Use of spirituous and fermented Liquors. Spices. Man by Nature not a drinking Animal.*

As almost all nations, that have deviated from the simple aliment of nature, unite to the use of animal food that of spirituous and fermented liquors, Dr. Lambe thinks it right not wholly to overlook the effects of these liquors on the human body. We find that the use of fermented liquors takes off the uneasy sensations of oppression, fulness, and nausea, which animal food, particularly fish, so frequently induces. This effect probably arises from the agreeable impression it makes on the stomach, counteracting the uneasiness caused by the solid aliment, rather than from any assistance it gives the digestion. We can determine little or nothing in the question of the salubrity or insalubrity of these liquors, from the agreeable or uneasy feelings which may immediately arise from them; or from the evidence and pretended experience of those that use them. The advocates for the use of fermented liquors are as worthy of credit as their opponents; as they must be supposed to give a faithful account of their own feelings. They will not be talked out of those feelings by the assertion, that the bodily strength furnished by beer, for instance, is only in proportion to the solid part of the barley dissolved in the water. We undoubtedly know, by experience, that the moderate use of fermented liquors augments, for a time, the muscular strength. This is, in fact, caused by the temporary stimulus they apply to the surface of the stomach, as all the muscles of the body sympathize with this membrane. But, to estimate the effects of these substances, we must consider the whole of their properties. The first is, that they diminish the appetite, by impairing the digestion. Unprincipled mothers give spirits to their infants, knowing it will lessen their appetite for bread. Fermented liquors, though they do not cause sleep as powerfully and certainly as opium, remarkably diminish the sensibility of the nervous system: they blunt the edge of the greatest evils the poor man suffers, hunger, weariness, and cold; he therefore flies for relief to spirits, regardless of their ultimate consequence. There appears also to be a permanent diminution of sensibility in persons habitually using fermented liquors. The spirit undergoes no change in the stomach; it is absorbed into the circulating mass: the body therefore is, in a greater or less degree, constantly under its influence. It was remarked by Savage, that the New Zealanders enjoyed a great superiority over the Europeans in sight and hearing. This superiority cannot be attributed to any other cause than their unacquaintance with fermented liquors, as they used flesh and fish as abundantly as Europeans. It appears then, that the advantages experienced from fermented liquors and from animal food are subject to the same limitation, and are regulated by the same laws. In diseases of debility, the stimulus of animal food and of fermented liquors may do no sensible injury, may even produce great apparent advantages: but in all constitutional diseases, in which there is a radical loss of power, this stimulation must impair the strength, and accelerate the fatal issue of the disease. The habitual use of fermented liquors will of itself counteract all the good effects of a wholesome diet, or of a salubrious situation. In the *Pays de Vaud,* in Switzerland, where drunkenness is particularly prevalent, half the population survive the period of forty years; scarcely a fourth part die in the first three years from their birth; but, notwithstanding these strong indications of general salubrity, after forty the probabilities of living decrease very fast; and after sixty-five they appear to be lower in this country than is common elsewhere. A state of impaired sensibility is not the consequence of the use of fermented liquors only: it is produced by the use of animal food likewise; as is evident from the improvement of the senses consequent on the relinquishing it for vegetable food only. As the putrescent matter of water is powerful enough to induce palsy, this substance must, evidently, have an analogous effect. We may conclude then, that fermented liquors, animal food, and impure water, injure the digesting and the secreting powers, and derange the other functions of the body. As then the diseases which are, with justice, ascribed to spirituous potations, often occur when this evil custom cannot be traced, it is reasonable to suspect that the liquors are not the sole agents, but only an accelerating and concurring cause in the production of these diseases.

A multitude of observations, which must occur to us all, give great probability to the opinion, that the use of fermented liquors occasions dropsy, epilepsy, palsy, insanity, and other the greatest calamities incident to human nature. Still, however, as these diseases cannot be warded off by the strictest temperance, they cannot be deemed the specific effect of the poison of alcohol, but the ultimate effect of various concurring morbific powers, acting on different persons according to the susceptibility and predisposition of each individual. That fermented liquors should be deleterious, is a proof of the wisdom and beneficence of the over-ruling Power. Were it otherwise, the rich would be enabled absolutely to starve the poor, by their wasteful consumption of the articles of first necessity. To make a pint of wine, at least three or four pounds of grapes are used; enough amply to support a man for a day. The man, therefore, who drinks his pint of wine daily, uses his own proper quantity of food, and destroys at the same time what might have been the food of another man. As the power of swallowing wine is almost unlimited, to what an extent would this mischief spread, if it did not find its natural boundary in the destruction of life which such habits occasion?

The action of this morbific agent is not the less real because it is slow, and the impression is, for a time, hardly perceptible. An adherence to this habit being borne with impunity, proves not the salubrity of the habit, but the flinty hardness of constitution with which some are blessed. However hot and fiery spices are in the mouth, they do not appear to be deleterious. They do not stupify the nervous system; they do not even greatly accelerate the pulse. The experience and opinions of Mr. Bruce, concerning these substances, are worthy of attention.

"I lay it down as a positive rule of health, that the warmest dishes the natives delight in, are the most wholesome strangers can use, in the putrid climates of Lower Arabia, Abyssinia, Senaar, and Egypt itself; and that spirits, and all fermented liquors, should be regarded as poisons." *Bruce's Travels*, vol. iv. p. 242.

"Having attempted to shew the noxious effects of water on the system; having condemned spirituous and fermented liquors, from the common experience of mankind, it must follow, that there is no species of drinking which I approve Indeed, I have already ventured to assert, that man is not naturally a drinking animal.* To those who cannot raise their views above the passing scene, this must appear a strange, a ridiculous assertion. We know very little about the habits of animals, except of those whose natures we have corrupted by domestication. We, however, know enough of the wild species to be certain, that the assertion of the necessity of the use of water to animals is, to the extent to which it is carried, absolutely groundless. Let us consider man fresh from the hands of his Maker, and depending on his physical powers only for his subsistence. Man has the head elevated above the ground, and to bring the mouth to the earth requires a strained and painful effort. Moreover, the mouth is flat, and the nose prominent; circumstances which make the effort still more difficult. He cannot even convey a fluid into his mouth without the aid of some artificial instrument. Nature seems, therefore, to have fully done her part towards keeping man from the use of liquids: and, doubtless, on a diet of fruits and recent vegetables, there would be no thirst, and no necessity for the use of liquids." 268.

We have thus given a fair view of the first part of Dr. Lambe's treatise; and, ere we proceed to the cases adduced in support of our author's doctrines, we shall make a few observations of our own.

---

* Reports on Cancer, p. 2.

It has been our lot to visit very many of those nations and tribes which Dr. Lambe brings forward as exhibiting examples of the influence of diet on the range of existence, and on the state of the constitution. Neither did we fail to keep an eye of inquiry on this very subject, though perfectly unacquainted with the investigations of our author at the time. The result of our observations was, that, whether we dine with the Ottomaque on clay, with the Englishman on beef, with the Aleutian on putrid whale, with the Irishman on potatoes, the Frenchman on soup, the Spaniard on grapes, the Italian on olives, or the Hindoo on rice, we shall, if *moderation* be observed, and other things be equal, attain the common limit of human existence; in short, that

—— Few simple foods,
Of all that earth, or air, or ocean yield,
But by *excess* offend.

Here, however, lies the point. There is scarcely a tribe on the face of the earth that does not commit excess in eating; and this excess is certainly more injurious in animal than in vegetable fare. Upon the whole, we are disposed to think, that man was designed by nature to be more a graminivorous than a carnivorous animal; but that he was never meant to be exclusively restricted to any one species in particular.

In respect to drink, we are perfectly of our author's opinion, that water is the only beverage which is necessary or wholesome; and we agree with him also, that if man lived on raw and esculent fruits or roots alone, he would probably never have occasion to drink at all. It is, therefore, more to be wished than expected, that man should relinquish the gratification of his palate for the prolongation of his life and the preservation of his health. But a very few of the human species will ever do this, and therefore it is needless to waste our time in the attempt to persuade them. The influence, however, of aqueous drink and vegetable food on the human constitution, both as preventive and curative of disease, is, in our opinion, still greater than Dr. Lambe imagines; and this subject shall come under review in our next Number.

